# Reproducibility of foot dimensions measured from 3-dimensional foot scans in children and adolescents with Down syndrome

**DOI:** 10.1186/s13047-020-00403-1

**Published:** 2020-06-04

**Authors:** Nirmeen M. Hassan, Andrew K. Buldt, Nora Shields, Karl B. Landorf, Hylton B. Menz, Shannon E. Munteanu

**Affiliations:** 1 Discipline of Podiatry, School of Allied Health, Human Services and Sport, La Trobe University, Victoria, 3086 Australia; 2 Living with Disability Research Centre, School of Allied Health, Human Services and Sport, La Trobe University, Victoria, 3086 Australia; 3 La Trobe Sport and Exercise Medicine Research Centre, School of Allied Health, Human Services and Sport, Victoria, 3086 Australia; 4 Discipline of Physiotherapy, School of Allied Health, Human Services and Sport, La Trobe University, Victoria, 3086 Australia

**Keywords:** Foot, Shoes, Down syndrome, Child, Adolescent, Foot deformities, 3-D image, Reproducibility of results

## Abstract

**Background:**

Children and adolescents with Down syndrome have a distinctive foot shape (such as wide and flat feet) that often leads to difficulty with footwear fitting. 3-dimensional (3D) scanning can accurately measure the foot dimensions of individuals with Down syndrome, which may assist shoe fit. However, the reproducibility of measuring foot dimensions using 3D scans in children and adolescents with Down syndrome is unknown. The aim of this study was to determine the intra- and inter-rater reproducibility of measuring foot dimensions of children and adolescents with Down syndrome using 3D scanning.

**Methods:**

3D foot scans of 30 participants with Down syndrome aged 5 to 17 years were obtained using the FotoScan 3D scanner. Foot dimensions assessed were foot length, ball of foot length, outside ball of foot length, diagonal foot width, horizontal foot width, heel width, ball girth, instep girth, first and fifth toe height, and instep height. Additionally, the Wesjflog Index and forefoot shape were determined. Measurements were completed by two raters independently on two separate occasions, 2 weeks apart. Intra- and inter-rater reliability were assessed using intra-class coefficients (ICCs) and Gwet’s AC1 statistics with 95% confidence intervals. Agreement was determined by calculating limits of agreement (LOA) and percentage agreement.

**Results:**

Eighteen participants were female and 12 were male (mean age 10.6 [3.9] years). Intra-rater reproducibility (ICCs ranged from 0.74 to 0.99, 95% LOA from − 13.7 mm to 16.3 mm) and inter-rater reproducibility (ICCs ranging from 0.73 to 0.99, 95% LOA from − 18.8 mm to 12.7 mm) was good to excellent, although some measurements (ball of foot length, outside ball of foot length, heel width and girth measurements) displayed wider LOAs indicating relatively poorer agreement. Forefoot shape displayed substantial to almost perfect reliability (Gwet’s AC1 0.68 to 0.85) and percentage agreement ranged from 73 to 87%, indicating acceptable agreement.

**Conclusions:**

The measurement of specific foot dimensions of children and adolescents with Down syndrome using 3D scans is reproducible. Findings of this study may be used to support future research measuring specific foot dimensions of children and adolescents with Down syndrome using 3D foot scans.

## Background

Down syndrome is the most common chromosomal disorder [1], occurring in 1 in every 650 to 1000 live births [2]. Down syndrome affects multiple body systems including the nervous, cardiovascular and the musculoskeletal systems [3], resulting in intellectual and physical disability. Individuals with Down syndrome can have reduced physical fitness [4], ligamentous laxity, hypotonia, reduced lower limb muscle strength [5], less functional gait patterns [6] and gait instability [7].

Children and adolescents with Down syndrome commonly experience conditions associated with the foot that may impact their physical function. A population-based study involving 197 young individuals with Down syndrome reported 63% of individuals with Down syndrome were affected by a musculoskeletal condition of the foot [8]. At present, the exact cause of many of these conditions is not clearly understood. However, a contributing factor may be the unique foot shape of this population leading to difficulties in finding appropriately-fitting footwear. Children and adolescents with Down syndrome often have a flatter, shorter and broader foot [9], and are more likely to have foot deformities that includes lesser toe deformities and hallux valgus [9]. These deviations in foot shape are likely to contribute to the number of children and adolescents with Down syndrome who wear poorly-fitting footwear; 60 to 88% of children and adolescents with Down syndrome [10, 11] compared to 16% of typically developing children [11]. Poorly-fitting footwear can have adverse outcomes including the development of foot pain, which may lead to impaired health-related quality of life and altered gait patterns [12, 13]. Additionally, poorly-fitting footwear may contribute to reduced physical activity in children and adolescents with Down syndrome [13]. This is an issue for children and adolescents with Down syndrome because they have low levels of physical activity and are at greater risk of developing chronic health conditions as a result. It may also contribute to their reduced social participation. Further, having an intellectual disability can be an additional complicating factor as children and adolescents with Down syndrome may not always verbally communicate pain experienced to their caregivers despite being more sensitive to pain [14].

Given the high prevalence of poorly-fitting footwear and the potentially detrimental effects on health, improving footwear-fit for children and adolescents with Down syndrome is important. Improving footwear fit may result in improved health-related quality of life and participation in physical activities. Additionally, improving the health of children and adolescents with Down syndrome may reduce burden to the health care system. Improving footwear fit can be achieved by designing footwear that can accommodate the unique foot shape of this population [12]. An essential initial step in designing footwear for children and adolescents with Down syndrome would be to capture the detailed foot dimensions of this population using reliable and valid methods. Although there is general consensus the foot shape of children and adolescents with Down syndrome differs to typically developing children, only one study [15] demonstrated the feet of young males with Down syndrome were shorter and narrower than age-matched peers, as measured using a podoscope. However, a limitation of this study is that only 2 foot dimensions (foot length and width) were measured using a 2-dimensional technique [8], which may not fully represent the complex shape of the foot. Three-dimensional (3D) scanning technology is a valid and reproducible means of obtaining detailed data on foot shape [16] and has been used to study the variations in foot shape in a number of different populations [17–19]. However, no studies have used 3D scanning to evaluate the foot dimensions of children and adolescents with Down syndrome, and the reproducibility of performing these measurements is unknown. Therefore, the aim of this study is to determine the reproducibility of measuring foot dimensions of children and adolescents with Down syndrome using 3D foot scanning.

## Methods

This study is reported in accordance with Guidelines for Reporting Reliability and Agreement Studies [20].

### Study design

Data were obtained from a previous feasibility study investigating the efficacy of custom-fitted footwear to increase physical activity levels in children and adolescents with Down syndrome [21]. The study was approved by the La Trobe University Human Ethics Committee (HEC16–027) and written informed consent was obtained from parents or guardians. Where appropriate, children and adolescents also provided written assent for participation prior to enrolment [21].

### Participants

Participants were children and adolescents aged 5 to 17 years with Down syndrome. Participants were excluded if they had any health condition that may affect physical activity (e.g. inflammatory arthritis, subluxation etc.) as reported by their parents, or required the use of an ambulatory device (e.g. cane, crutches or walker). Participants were recruited through a member-based disability organisation for individuals with Down syndrome that was based in the community [21].

### Raters

Two raters (NMH and AKB) performed the measurements for all foot scans, and measurements were repeated twice within 4 weeks to assess intra- and inter-rater reproducibility. Both raters were registered podiatrists with four and 14 years of clinical experience, respectively. Rater 1 had 3 months and rater 2 had 5 years of experience using 3D scans to measure foot dimensions, respectively.

### Measurements of participant characteristics

Participants had their height and weight measured to calculate body mass index (BMI). Measurements were for the right foot only. Foot posture was assessed using two indices; the Foot Posture Index [22] and the Arch Index [23]. For both indices, higher positive scores indicate a flatter foot posture. The presence of lesser digital deformities (i.e. hammer, mallet and claw toes) was documented [10, 24]. The presence and severity of hallux valgus deformity was assessed using the Manchester scale [25]. The degree of deformity was graded on a scale of 0 to 3 (no deformity, mild, moderate and severe). Scores for hallux valgus were dichotomised, where scores of 0 and 1 were graded as absent and scores of 2 and 3 were graded as present [26].

### Scanning procedure

**Fig. 1:**
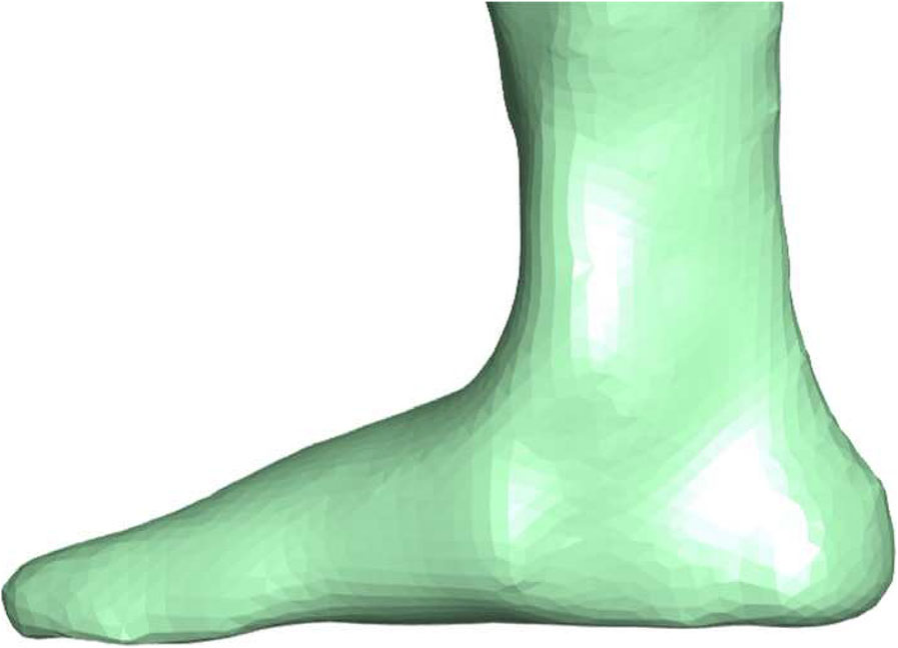
3D model of right foot viewed from the medial aspect

Participants stood in a relaxed, full weight-bearing position and a 3D scan was taken of their right foot using the FotoScan 3D scanner (Precision 3D, Weston-super-mare, UK). The FotoScan 3D device uses a fixed system of cameras and projectors to obtain images of the foot, that are automatically converted into a 3D model [27]. According to the manufacturer, the scans obtained with this system are accurate to within less than half a millimetre. The 3D foot scans were then exported as stereolithography (STL) files (Fig. 1). The 3D-Tool© Version 13 (3D-Tool GmbH, Weinheim, Germany) was used to obtain all length, width and height measurements. For girth measurements, a cross-section of the foot at the relevant landmarks was created and exported as a drawing exchange format file. The perimeter of the cross-section was determined using Canvas© 11 software (ACD Systems International, Seattle, WA, USA).

### Measurement of foot dimensions

Prior to data collection, pilot testing of the foot measurements was completed. A measurement technique protocol was developed (see Additional file 1). The protocol was piloted on the scans of five participants and the results were compared. Where there was substantial variability in results, the measurement technique was clarified between raters until consensus was reached. Each rater worked independently during data collection and compiled measurements on separate Excel spreadsheets (Microsoft® Office 365, Microsoft, Redmond, WA, USA) on two separate occasions that were two weeks apart.

**Fig. 2:**
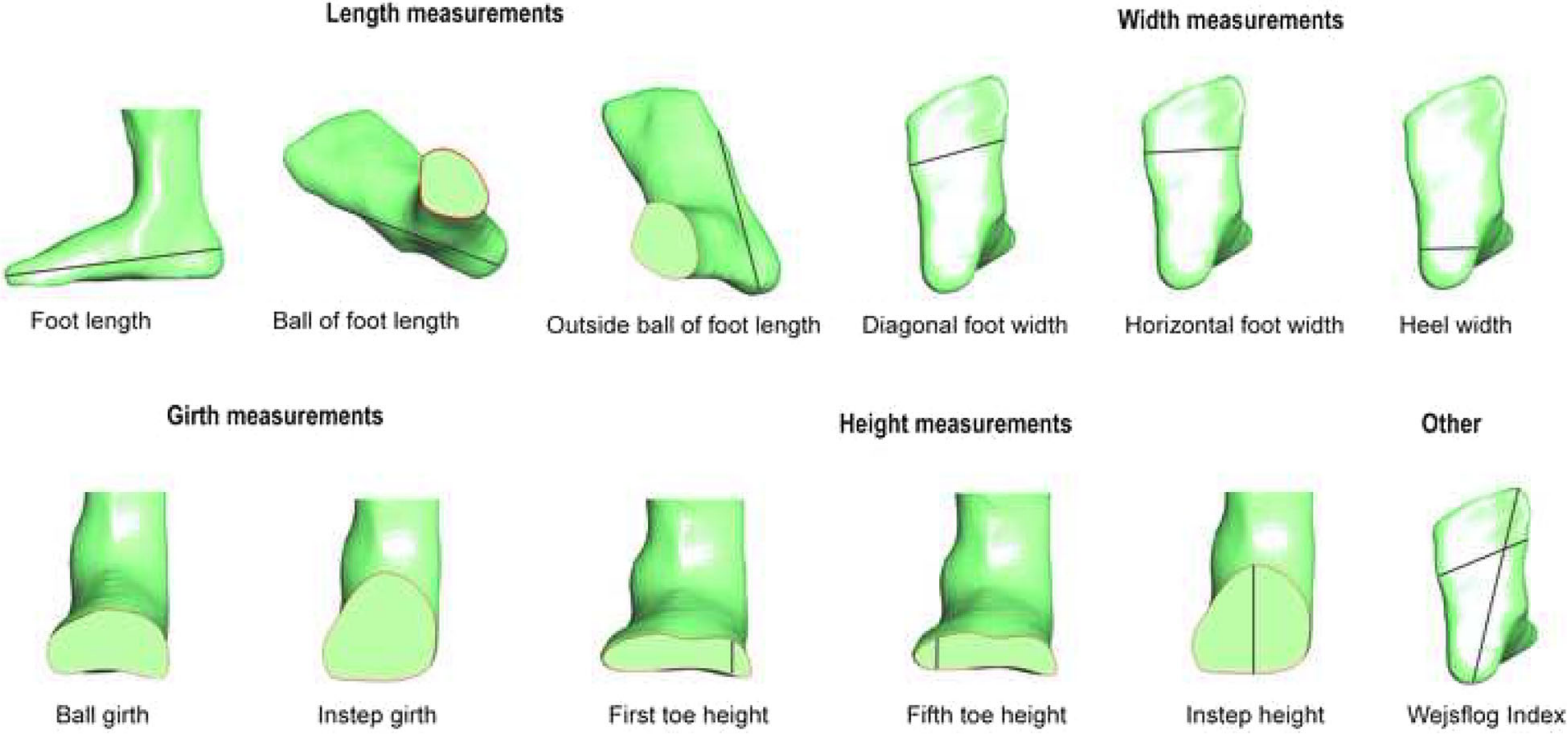
Foot measurements

**Fig. 3:**
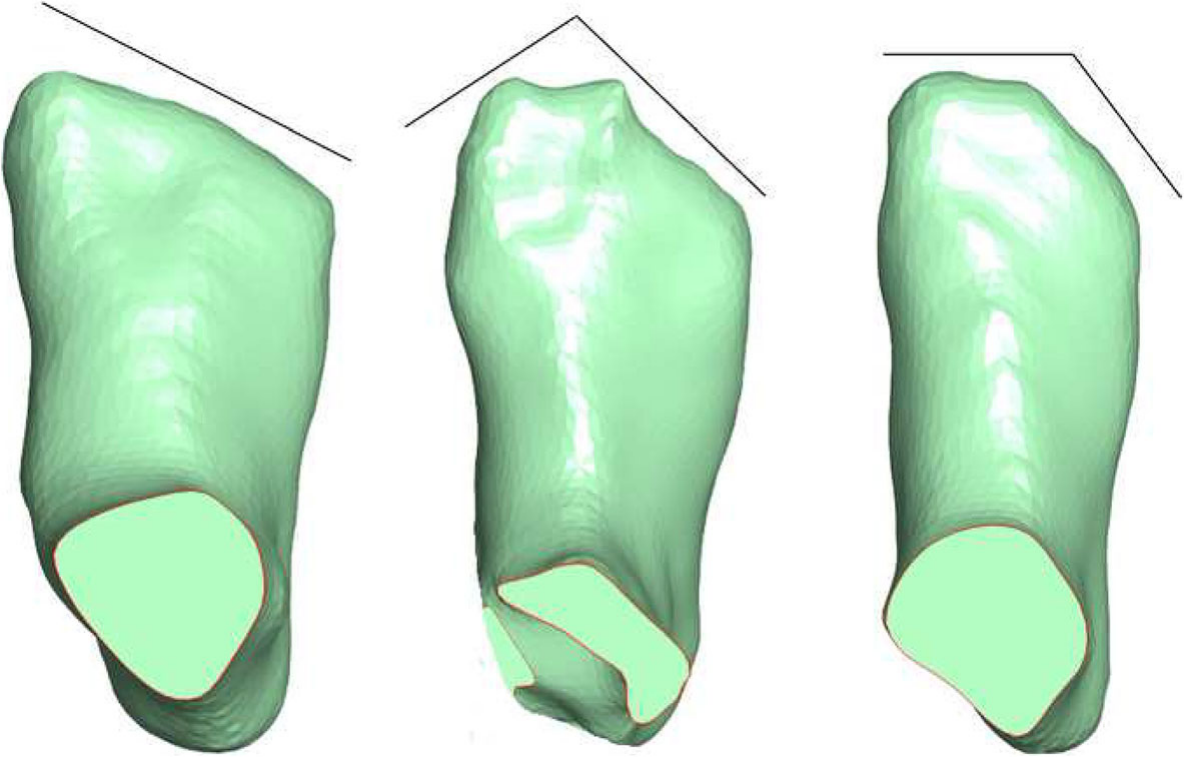
Measurements of forefoot shape: **a** first toe longest; **b** second toe longest; and **c** first and second toe length equal

We measured 13 foot dimensions that are relevant to footwear manufacturing [17, 28, 29]. These were (Figs. 2 and 3):

1. Foot length: distance between foot end (pternion) and foot tip (anterior point of most protruding toe).
2. Ball of foot length: distance between foot end (pternion) and the first metatarsophalangeal protrusion.
3. Outside ball of foot length: distance between foot end (pternion) and the fifth metatarsophalangeal protrusion.
4. Diagonal foot width: connecting line between the first metatarsophalangeal joint and the fifth metatarsophalangeal joint.
5. Horizontal (orthogonal) foot width: orthogonal connection line starting at the first metatarsophalangeal joint to the outside curvature of the foot.
6. Heel width: maximum, orthogonal connection line starting at the medial side of the heel to the outside curvature of the heel.
7. Wejsflog Index: a ratio measurement of foot length to the diagonal forefoot width.
8. Ball girth: maximum circumference at the level of the first and the fifth metatarsophalangeal joint protrusion.
9. Instep girth: maximum circumference measured from the most plantar aspect of the foot to the most dorsal aspect of the foot, at the level of the navicular.
10. First toe height: maximum height of the hallux measured from the most plantar aspect of the hallux to the most dorsal aspect of the hallux.
11. Fifth toe height: maximum height of the fifth toe measured from the most plantar aspect of the fifth toe to the most dorsal aspect of the fifth toe.
12. Instep height: measured from the most plantar aspect of the foot to the most dorsal aspect of soft tissue (plantar foot end to the junction of shank and foot dorsum).
13. Forefoot shape: determined by categorising forefoot shape into three categories using the length of the digits. The categories were (i) first toe longest; (ii) second toe longest; and (iii) first and second toe length equal length.

### Statistical analysis

An a priori sample size estimation using the Power Analysis and Sample Size software (PASS 15 software, NCSS, LLC, Kaysville, UT, USA) for the ICC test, alpha of 0.05, power 0.80 and ICC of 0.60 determined that a minimum sample size of 19 participants (foot scans) was necessary. However, as we had access to foot scans of 30 children and adolescents with Down syndrome, all scans were measured. Analyses were performed using IBM SPSS Statistics version 25.0 (IBM Corp, NY, USA) and STATA SE Version 14.2 (StataCorp, College Station, TX, USA), using the *kappaetc* module. To satisfy the independence assumption of statistical analysis, measurements from the right foot only were analysed [30]. For continuous data, data were assessed for normality using skewness and kurtosis tests, and data were found to be normally distributed. For continuous data, reliability was calculated using ICCs with 95% confidence intervals (ICC [1, 2], consistency) [31]. Interpretation of ICCs were based on definitions provided by Portney and Watkins [32], where ICC values less than 0.5 was considered as poor, values between 0.5 to 0.75 was considered as moderate, values between 0.75 to 0.90 was considered as good and any value above 0.90 was considered as excellent reliability. For nominal data, reliability was determined by calculating Gwet’s AC1 statistic [33] and results were interpreted according to Landis and Koch cut-offs, which are: less than zero was considered poor, 0 to 0.20 slight, 0.21 to 0.40 fair, 0.40 to 0.60 moderate, 0.60 to 0.80 substantial, 0.81 to 1.00 almost perfect reliability [34]. For continuous data, agreement was determined by calculating limits of agreement (LOA) [35]. For the calculation of LOAs, the presence of heteroscedasticity was assessed, and when present, ratio LOAs were calculated by taking the antilog of the calculated LOA values [36]. For nominal data, agreement was determined by using percentage agreement.

## Results

Table 1 summarises the participant characteristics. There were 18 females and 12 males, with a mean (SD) age of 10.6 (3.9) years. The mean Foot Posture Index was + 9 (1.8) and the mean Arch Index was 0.30 (0.05), indicating that on average, participants had a flat foot type. Hallux valgus was present in 4 participants (13%), and 14 participants (46%) had some degree of deformity of one of the lesser toes. No participants were excluded on the basis of requiring an ambulatory device.

**Table 1:** Participant characteristics – values are mean (SD) unless otherwise stated

Characteristic	Mean (SD)
Age, years	10.6 (3.9)
Females/males, n	18/12
Height (m)	1.30 (0.19)
Weight (kg)	40.1 (21.4)
BMI^1^ (kg/m^2^)	21.9 (6.7)
Type of Down syndrome, n (Trisomy 21 / Translocation)	27/3
Level of intellectual disability (unclear/mild/moderate/severe)	9/12/9/0
Presence of hallux valgus, n (%)	4 (13.0)
Presence of digital deformity, n (%)	14 (46.0)
Foot Posture Index^2^	8.8 (1.8)
Arch Index^3^	0.30 (0.05)

^1^Body mass index; ^2^Foot Posture Index; ^3^Arch Index. The Foot Posture Index scores range from −12 to + 12. Scores less than 1 indicate a supinated foot posture, scores between 1 and 7 indicate a normal foot posture, and scores greater than 7 indicate a pronated foot posture. Arch Index represents the ratio of the area of the middle third of a footprint to the entire footprint area, excluding the digits. Normal: 0.21 to 0.28, high: < 0.21 and low: > 0.28

### Intra-rater reproducibility

Table 2 summarises the intra-rater reproducibility of all 13  foot measurements. The intra-rater reliability of rater 1 was good to excellent for 11 out of 13 measurements, with ICCs ranging from 0.76 to 0.99. Fifth toe height and forefoot shape measurements had moderate reliability only (ICC = 0.74, Gwet’s AC1 = 0.68 respectively). For rater 2, intra-rater reliability was good to excellent for all 13 measurements (ICCs/Gwet’s AC1 ≥ 0.83).

**Table 2:** Intra-rater reproducibility of foot measurements

	Rater 1				Rater 2			
	Trial 1	Trial 2			Trial 1	Trial 2		
Foot measurement	**Mean (SD)**	**Mean (SD)**	**ICC (95% CI) [Interpretation]**	**95% LOA**^**1**^	**Mean (SD)**	**Mean (SD)**	**ICC (95% CI) [Interpretation]**	**95% LOA**^**1**^
Foot length	193.3 (27.9)	192.7 (28.1)	0.99 (0.99 to 1.00) [Excellent]	−2.0 to 3.2	193.2 (27.6)	192.5 (28.0)	0.99 (0.99 to 1.00) [Excellent]	−1.6 to 3.0
Ball of foot length	146.0 (21.1)	145.6 (22.2)	0.97 (0.94 to 0.99) [Excellent]	−9.7 to 10.6	147.3 (21.0)	149.2 (21.9)	0.98 (0.97 to 0.99) [Excellent]	−8.7 to 4.8
Outside ball of foot length	127.5 (18.4)	124.9 (17.7)	0.97 (0.94 to 0.99) [Excellent]	−5.7 to 10.7	130.5 (15.8)	129.3 (18.0)	0.89 (0.80 to 0.95) [Good]	−13.7 to 16.3
Diagonal foot width	79.4 (13.1)	79.0 (13.0)	0.99 (0.98 to 1.00) [Excellent]	−3.2 to 3.9	78.4 (12.0)	78.4 (12.3)	0.98 (0.98 to 0.99) [Excellent]	−3.8 to 3.7
Horizontal foot width	77.3 (12.7)	77.1 (12.2)	0.97 (0.94 to 0.99) [Excellent]	−5.8 to 6.1 / 0.934 to 1.06^2^	76.8 (12.0)	76.9 (11.7)	0.98 (0.98 to 1.00) [Excellent]	−3.6 to 3.3
Heel width	48.4 (8.5)	49.4 (7.9)	0.76 (0.60 to 0.90) [Good]	−12.1 to 10.1	47.1 (8.6)	45.5 (7.6)	0.83 (0.70 to 0.91) [Good]	−7.7 to 10.8 / 0.86 to 1.23^2^
Wejsflog Index	2.5 (0.2)	2.5 (0.2)	0.96 (0.92 to 0.98) [Excellent]	−0.1 to 0.1	2.5 (0.21)	2.5 (0.21)	0.95 (0.90 to 0.98) [Excellent]	−0.1 to 0.1
Ball girth	190.3 (29.1)	190.6 (31.5)	0.98 (0.96 to 0.99) [Excellent]	−11.4 to 10.7	189.4 (29.6)	189.2 (30.7)	0.98 (0.97 to 0.99) [Excellent]	−9.8 to 10.3
Instep girth	202.5 (26.5)	204.4 (27.5)	0.97 (0.95 to 0.99) [Excellent]	−13.5 to 3.7	200.0 (27.5)	198.7 (26.9)	0.99 (0.98 to 1.00) [Excellent]	−5.8 to 8.3
First toe height	20.1 (4.5)	20.7 (3.6)	0.88 (0.80 to 0.94) [Good]	−4.3 to 3.3	20.7 (4.1)	21.2 (3.8)	0.83 (0.70 to 0.92) [Good]	−4.9 to 3.9
Fifth toe height^3^	16.93 (2.6)	17.6 (2.7)	0.74 (0.52 to 0.87) [Moderate]	−4.3 to 3.0	17.5 (2.8)	17.49 (2.6)	0.84 (0.70 to 0.92) [Good]	−2.9 to 2.9
Instep height	57.1 (7.7)	54.4 (8.0)	0.93 (0.90 to 0.97) [Excellent]	2.8 to 8.1	53.6 (7.5)	55.3 (6.7)	0.95 (0.91 to 0.98) [Excellent]	−5.7 to 2.5 / 0.89 to 1.05^2^
Forefoot shape	–	–	0.68 (0.45 to 0.90)^4^ [Substantial]	73 (60 to 90)^5^	–	–	0.85 (0.70 to 1.0)^4^ [Almost perfect]	87 (70 to 100)^5^

^1^Limits of agreement. ^2^Ratio limits of agreement also presented as measurement displays heteroscedasticity. ^3^29 scans were used due to the presence of artefacts. ^4^Gwet’s AC1. ^5^Percentage agreement. Foot dimensions are measured in millimetres

There were 6 measurements (foot length, diagonal foot width, Wejsflog Index, first toe height, fifth toe height and instep height) for both raters and 1 measurement for Rater 2 (horizontal foot width) that exhibited narrow LOAs ranging from − 5.7 mm to 8.1 mm. However, 5 measurements (ball of foot length, outside ball of foot length, heel width, ball girth, and instep girth) demonstrated wider LOAs ranging from − 13.5 mm to 16.3 mm. For forefoot shape, agreement ranged from 73 to 87%.

### Inter-rater reproducibility

Table 3 summarises the inter-rater reproducibility of all 13 foot measurements. The inter-rater reliability was good to excellent for all 11 measurements, with ICCs ranging from 0.89 to 0.99. Fifth toe height and forefoot shape measurements had moderate reliability only (ICC = 0.73 and Gwet’s AC1 = 0.77, respectively).

**Table 3:** Inter-rater reproducibility of foot measurements

Foot measurement	Rater 1 Mean (SD)	Rater 2 Mean (SD)	ICC (95% CI) [Interpretation]	95% LOA^1^
Foot length	193.3 (27.9)	193.2 (27.6)	0.99 (0.99 to 1.00) [Excellent]	−2.8 to 2.9 / 0.99 to 1.01^2^
Ball of foot length	146.0 (21.1)	147.3 (21.0)	0.97 (0.94 to 1.00) [Excellent]	−10.8 to 8.3
Outside ball of foot length	127.5 (18.4)	130.5 (15.8)	0.89 (0.80 to 0.95) [Good]	−18.8 to 12.7
Diagonal foot width	79.4 (13.1)	78.4 (12.0)	0.98 (0.96 to 0.99) [Excellent]	−3.8 to 5.8
Horizontal foot width	77.3 (12.7)	76.8 (12.0)	0.98 (0.96 to 0.99) [Excellent]	−4.3 to 5.4
Heel width	48.4 (8.5)	47.1 (8.6)	0.91 (0.81 to 0.95) [Excellent]	−5.9 to 8.6 / 0.89 to 1.19^2^
Wejsflog Index	2.4 (0.2)	2.5 (0.2)	0.90 (0.82 to 0.96) [Good]	−0.2 to 0.2
Ball girth	190.3 (29.1)	189.4 (29.6)	0.99 (0.97 to 0.99) [Excellent]	−8.5 to 10.2
Instep girth	202.5 (26.5)	200.0 (27.5)	0.98 (0.97 to 0.99) [Excellent]	−7.3 to 12.2
First toe height	20.1 (4.5)	20.7 (4.1)	0.92 (0.84 to 0.96) [Excellent]	−3.9 to 2.9
Fifth toe height^3^	16.9 (2.6)	17.5 (2.8)	0.73 (0.50 to 0.86) [Moderate]	−4.4 to 3.3
Instep height	57.1 (7.7)	53.6 (7.4)	0.90 (0.81 to 0.95) [Good]	−3.0 to 9.9
Forefoot shape	–	–	0.77 (0.58 to 0.96)^4^ [Substantial]	80 (65 to 95)^5^

^1^Limits of agreement. ^2^Ratio limits of agreement also presented as measurement displays heteroscedasticity. ^3^29 scans were used due to the presence of artefacts. ^4^Gwet’s AC1. ^5^Percentage agreement. Foot dimensions are measured in millimetres

Six measurements (foot length, diagonal foot width, horizontal foot width, Wejsflog Index, first toe height and fifth toe height) demonstrated narrow LOAs, ranging from − 4.4 mm to 5.8 mm. However, the remaining 5 measurements (ball of foot length, outside ball of foot length, ball girth, instep girth and instep height) displayed relatively wider LOAs ranging from − 18.8 mm to 12.7 mm. Percentage agreement for forefoot shape was 80%.

## Discussion

We found that the foot dimensions of children and adolescents with Down syndrome can be measured reliably from 3D foot scans. Our results indicate moderate to excellent reliability for all foot dimension measurements as demonstrated by high inter-rater ICC values or Gwet’s AC1 values. However, the measurement of fifth toe height displayed poorer reliability. We observed some measurements (foot length, diagonal foot width, horizontal foot width, Wejsflog Index, first toe height and fifth toe height) had narrow LOAs indicating good agreement between raters, whereas others (ball of foot length, outside ball of foot length, ball girth, instep girth and instep height) displayed wider LOAs, suggesting relatively poorer agreement.

We observed differences in the performance of the two raters. The reliability for rater 1 was poorer than rater 2 for the measurements of heel width, fifth toe height and forefoot shape. The reason for this is unclear but could be that these measurements are more challenging to measure due to difficulty in locating the boundaries of these regions, particularly the toe region [16]. It is also possible that varying experience of the raters influenced the findings, since the reproducibility of rater 2, who had 5 years of experience, was greater than rater 1, who had 3 months’ experience. This speculation is supported by previous work that has shown that rater experience and training is an important consideration in the reliability of measuring foot dimensions, particularly when it involves manual allocation of landmarks for calculating dimensions [16].

As this is the first study to investigate the reproducibility of the measurement of foot dimensions of children and adolescents using 3D scans, it is not possible to directly compare our findings. However, our findings are in general agreement with studies that investigated the reliability of measuring foot dimensions using adult foot scans where ICCs ranged from 0.82 to 0.99 [16, 27, 37–40].

Our findings relating to agreement allows for the interpretation of the acceptability of the reproducibility of the measurements. Where the LOA value is less than the minimally important difference for a measurement, the agreement could be considered acceptable. However, the value for the minimally important difference may vary depending on the context of the measurement. For example, for footwear fitting, it is common practice that foot length and foot width are measured in order to select an appropriately sized shoe. The International Organization for Standardization (2015) for footwear reports whole sizes (US and UK) differ in length by 8 mm [41], which indicates the reproducibility of the measurement of foot length is likely to be acceptable, as the range for the inter-rater LOA value for this measurement was 3.9 mm. That is, it is less than the value that would necessitate a difference in shoe size (8 mm). In contrast, the acceptability of the measurement of horizontal foot width is questionable, as the range of the inter-rater LOA value was 9.7 mm, which exceeds the standard 4.8 mm that defines a difference in shoe width [42]. The acceptability of the reproducibility of the remaining measurements (if used to guide the manufacture of footwear) for children and adolescents with Down syndrome is unclear, as shoe last dimensions for different shoe sizes is commercially sensitive information and not readily available.

Footwear-fit can be difficult in children and adolescents with Down syndrome because of variations to the shape of their feet. This study has shown measuring the foot dimensions from 3D foot scans of children and adolescents with Down syndrome can be done reproducibly. As they are reproducible, these measurements can be used with confidence in several settings. In a clinical setting, clinicians could perform measurements of foot dimensions to monitor and inform parents of their child’s foot shape, and to provide guidance on appropriate footwear selection. In an applied research setting, the measurements may be used to determine detailed differences in foot dimensions of children and adolescents with Down syndrome, which may then be considered when manufacturing footwear for this population.

Our findings need to be interpreted in the context of the strengths and limitations of this study. We used two raters who worked independently throughout the data collection process in order to reduce the risk of bias, and we assessed both intra- and inter-rater reproducibility for completeness of results. In addition, both raters were podiatrists, who were experienced in foot and ankle measurements. However, the generalisability of the findings to raters from other professional backgrounds and/or with different experience in measuring 3D foot scans needs further investigation. Finally, the measurements in this study were manually calculated according to definitions that were outlined in our methods, so findings may differ if dimensions are calculated by scanning software with pre-populated definitions of measurements that are different to the ones that we used.

## Conclusions

The measurement of specific foot dimensions of children and adolescents with Down syndrome using 3D scans is reproducible. The measurement described has multiple applications. In a clinical setting, clinicians can perform measurements of foot dimensions to monitor and inform parents of their child’s foot shape, and to provide guidance on appropriate footwear selection. In an applied research setting, the measurements may be used to determine detailed differences in foot dimensions of children and adolescents with Down syndrome, which may then be considered when manufacturing footwear for this population.

## Supplementary information

**Additional file 1.** 3D foot scan measurement protocol

## Data Availability

The datasets used and/or analysed during the current study are available from the corresponding author upon reasonable request.

## Abbreviations

2D: 2-dimensional
3D: 3-dimensional
BMI: Body mass index
ICC: Intra-class coefficient
LOA: Limits of agreement
