# Correction to: Reproducibility of foot dimensions measured from 3-dimensional foot scans in children and adolescents with Down syndrome

**DOI:** 10.1186/s13047-020-00409-9

**Published:** 2020-07-02

**Authors:** Nirmeen M. Hassan, Andrew K. Buldt, Nora Shields, Karl B. Landorf, Hylton B. Menz, Shannon E. Munteanu

**Affiliations:** 1 Discipline of Podiatry, School of Allied Health, Human Services and Sport, La Trobe University, Victoria, 3086 Australia; 2 Living with Disability Research Centre, School of Allied Health, Human Services and Sport, La Trobe University, Victoria, 3086 Australia; 3 La Trobe Sport and Exercise Medicine Research Centre, School of Allied Health, Human Services and Sport, Victoria, 3086 Australia; 4 Discipline of Physiotherapy, School of Allied Health, Human Services and Sport, La Trobe University, Victoria, 3086 Australia

**Correction to: J Foot Ankle Res 13, 31 (2020)**

**https://doi.org/10.1186/s13047-020-00403-1**

Following publication of the original article [1], we have been notified of the below corrections:

1. On Page 1, Abstract, ‘Methods’ section, line 6: ‘intra-class coefficients’ should read ‘intra-class correlation coefficients’. This error is also present on page 8 in the ‘Abbreviations’ section.
2. In Table 1 on page 5, within the final sentence of the notes section at the bottom of the table: the cut-off values used for the Arch Index ‘Normal: 0.21 to 0.28, high: < 0.21 and low: > 0.28’, should read ‘Normal: 0.21 to 0.26, high: < 0.21 and low: > 0.26 [23]’

## References

[1] Hassan (2020). Reproducibility of foot dimensions measured from 3-dimensional foot scans in children and adolescents with Down syndrome. J Foot Ankle Res.

