# Supplementary material for: Reproducibility of foot dimensions measured from 3-dimensional foot scans in children and adolescents with Down syndrome
Source: J Foot Ankle Res. 2020 Jun 4;13:31. doi: 10.1186/s13047-020-00403-1 (PMC7271427; doi:10.1186/s13047-020-00403-1)
Supplement: Supplementary file 1 — Additional file 1. 3D foot scan measurement protocol [file 13047_2020_403_MOESM1_ESM.docx]

# **3D foot scan measurement protocol**

# Software required

- The 3D-Tool^©^ Version 13 (3D-Tool GmbH, Weinheim, Germany): <https://www.3d-tool.com/>
- Canvas^©^ 11 software (ACD Systems International, Seattle, WA, USA).

# Notes

- All length and width measurements can be measured using 3D-Tool^©^ viewer
- All girth measurements or cross-sections taken will require Canvas^©^
- Unit of measurement for all dimensions are in mm (both software)

# Length

## Foot length

**Definition**

Distance between foot end (heel) and foot tip (anterior point of most protruding toe).

**Instructions**


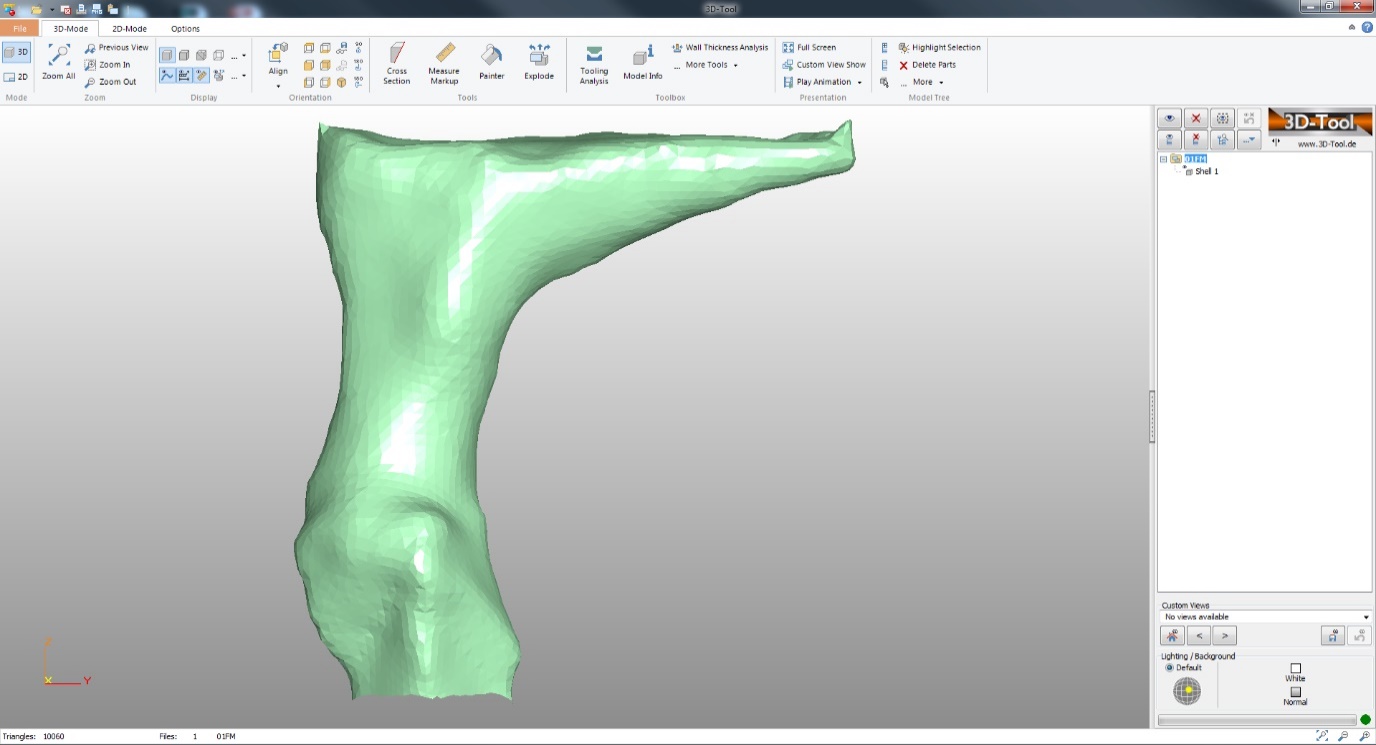

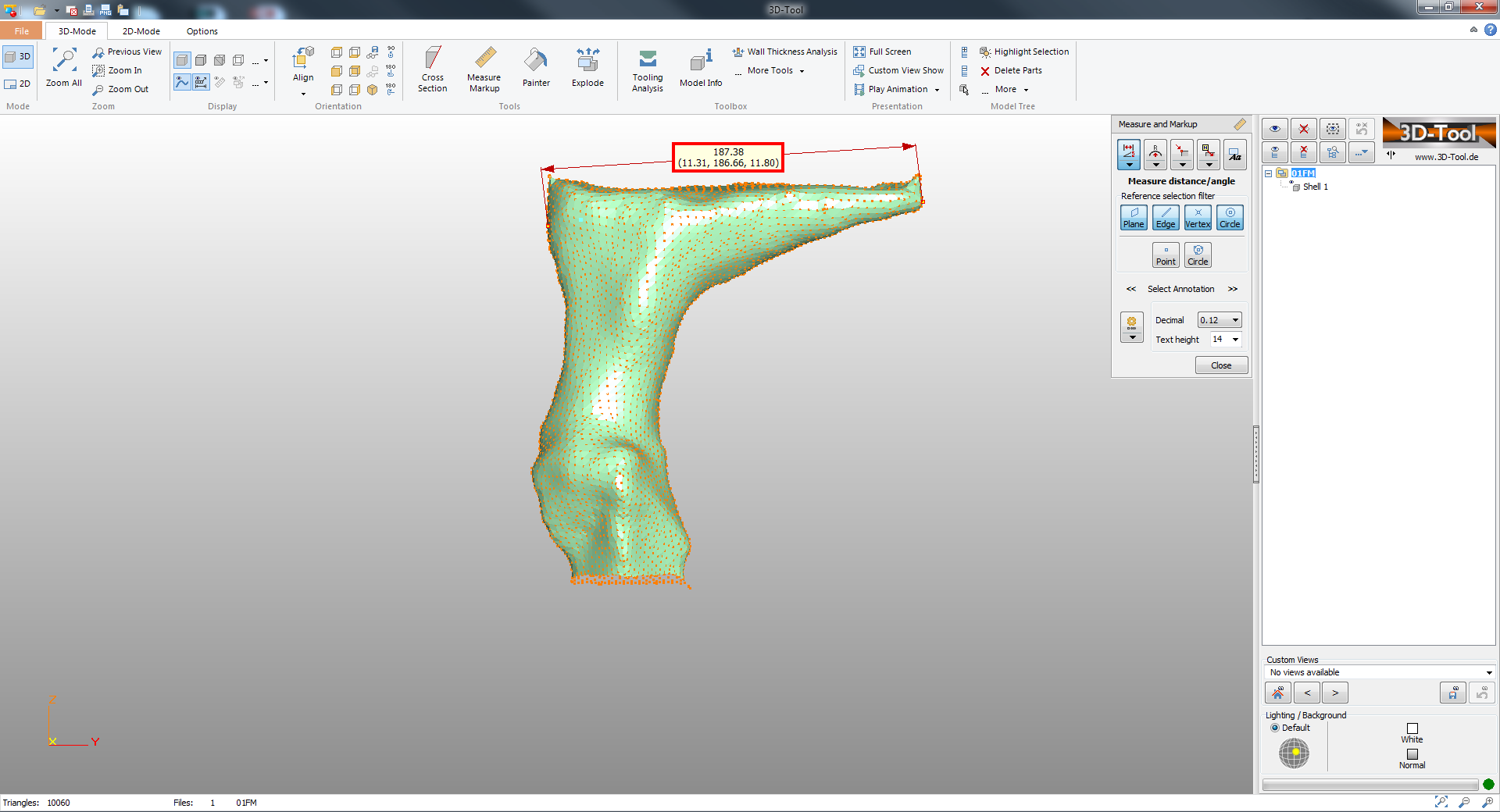


To measure length, click on right view under the Align tab. Select measure mark-up and select your landmarks from the pternion to the most protruding toe.

##
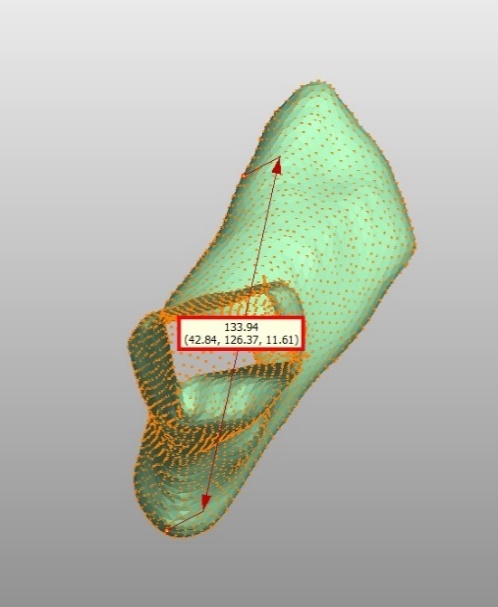
Ball of foot length

**Definition**

Distance between foot end (heel/pternion) and the 1^st^ metatarsophalangeal protrusion.

**Instructions**

To measure ball of foot length, rotate the 3D model to view the medial side of the foot. Select ‘measure mark-up’ and click on the heel from the pternion. Rotate the image to view the planter surface of the model. Select the landmark at the protrusion of the 1^st^ metatarsal. Rotate the model to check the dorsal side of the foot to confirm positioning (see image).

##
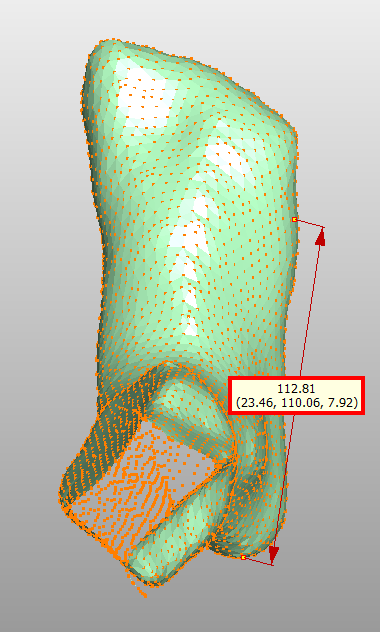
Outside foot length

**Definition**

Distance between foot end (heel/pternion) and the fifth metatarsophalangeal protrusion.

**Instructions**

Select the same point of the pternion used for foot length and ball of foot length, rotate the model to view the plantar side. Select the point of the protrusion of the 5^th^ metatarsal. Rotate the model to check the dorsal side of the foot to confirm positioning (see image).

# Foot width


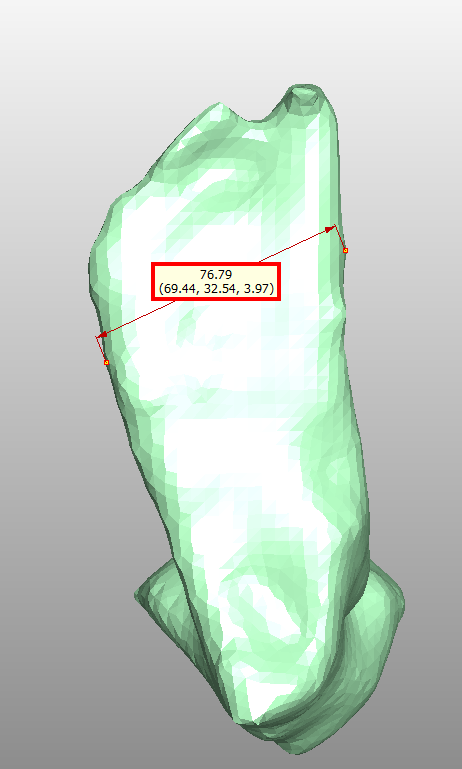

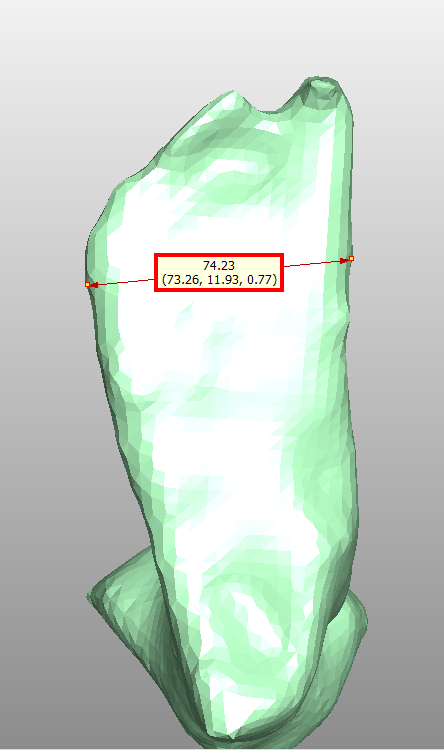

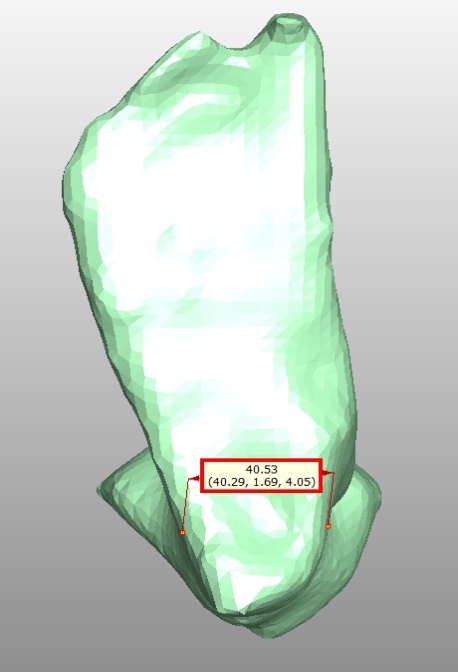


(A) (B) (C)

## Diagonal foot width

**Definition**

Connecting line between the 1^st^ metatarsophalangeal joint and the 5^th^ metatarsophalangeal joint.

**Instructions**

Position to view the plantar surface of the foot. Select “measure mark-up” and measure the widest points of the forefoot, at the 1^st^ metatarsal to the 5^th^ metatarsal, following the height of the metatarsal heads.

## Horizontal foot width

**Definition**

Orthogonal connection line starting at the 1^st^ metatarsophalangeal joint to the outside curvature of the foot.

**Instructions**

Similarly, select the 1^st^ metatarsophalangeal joint and measure to the outside curvature of the foot.

## (C) Heel width

**Definition**

Orthogonal connection line starting on the medial side of the heel to the outside curvature of the heel.

**Instructions**

Measure the widest part of the heel from the medial to lateral aspect of the heel.

# Girth

##
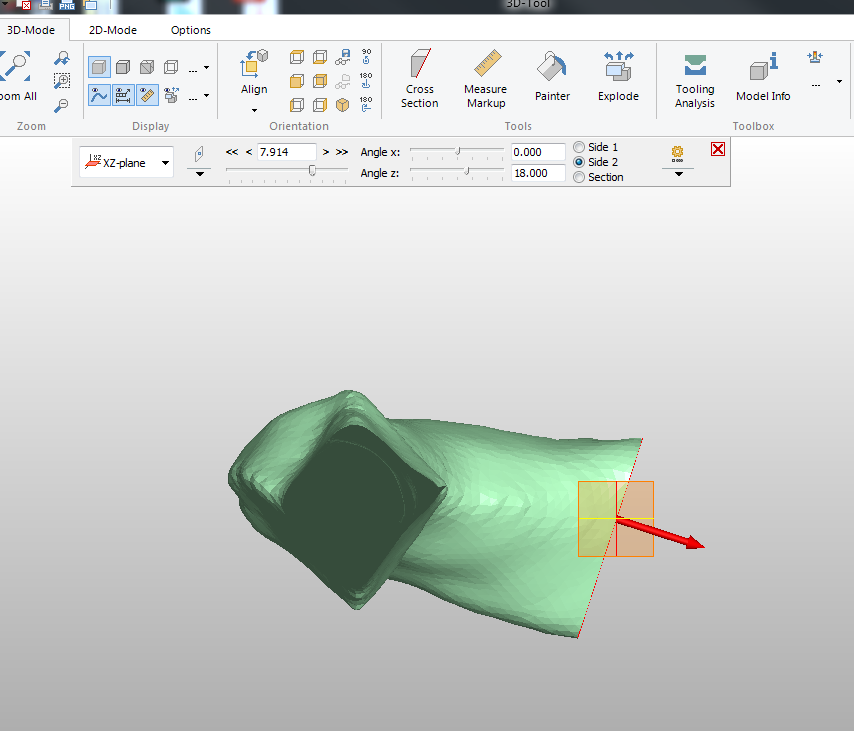
Ball girth

**Definition**

Maximum circumference over the first to the fifth metatarsophalangeal joint protrusion.

**Instructions**

Rotate the model to view the dorsal side of the foot. Select ‘cross section’. Select the ‘XZ-plane’ and drag the arrow to the ball of the foot. Adjust the ‘angle Z’ to follow the metatarsal head positioning. Select the ‘setting button’, select ‘export cross-section as DXF’ and save. You will need to open this file in Canvas^©^ to measure ball girth.

##
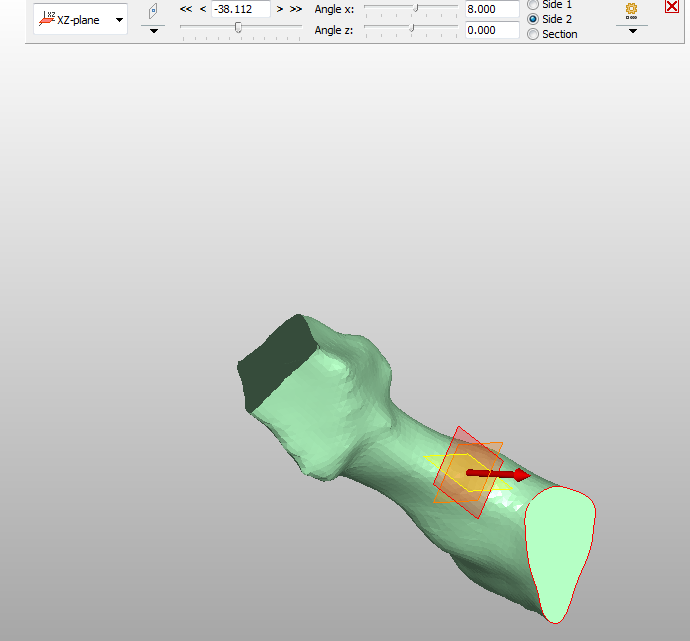
Instep girth

**Definition**

Measured from the most plantar surface of the foot to the most dorsal aspect of the foot, in alignment with the navicular.

**Instructions**

Similar to ball girth, drag the arrow to the instep region and adjust the angle to align with the navicular. Rotate the image for the best view of the final area, then export as a DXF to open and measure in Canvas^©^. For all girth measurements that are exported as a DXF and opened in Canvas^©^, a dialogue box will allow you to select from a range of options. Ensure settings are in mm, open the file and select the image. The top panel will show the perimeter value.

# Height

##
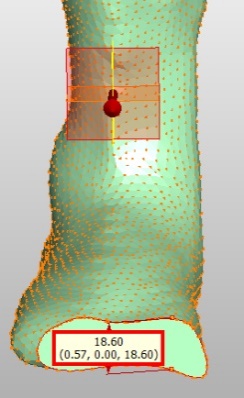
First and fifth toe height

**Definition (first toe height)**

Maximum height of the hallux measured from the most plantar aspect of the hallux to the most dorsal aspect of the hallux.

**Instructions**

Position the model to view the medial side. Take a cross-section of the highest point of the digit, reposition the foot model (to view from the front) and measure height.


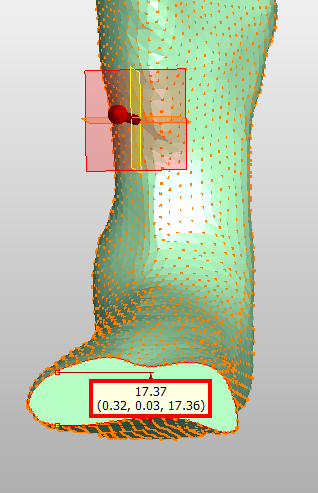
**Definition (fifth toe height)**

Maximum height of the 5^th^ toe measured from the most plantar aspect of the toe to the most dorsal aspect of the toe.

**Instructions**

Position the model to view the lateral side. Take a cross-section of the highest point of the digit, reposition the foot model (to view from the front) and measure height.

##
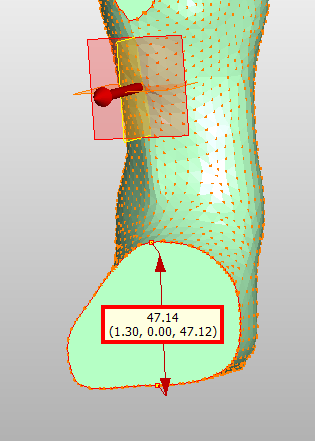
Instep height

**Definition**

Measured from the most plantar aspect of the foot to the highest dorsal aspect of soft tissue (plantar foot end to the junction of shank and foot dorsum).

**Instructions**

Rotate the model to view the medial side of the foot. Select ‘cross section’. Select the ‘XZ-plane’ and drag the arrow to the region according to the definition. With this cross-section of the instep height, measure from the most plantar aspect to the most dorsal aspect of soft tissue.

# Forefoot shape

The forefoot region of each scan has been categorised into 3 shapes, which reflect the length of toes relative to each other. This section involves evaluating the forefoot and determining which category best suits the shape of the toes for each scan.

Shapes are:

1. 1^st^ digit is the longest digit (1>2>3>4>5)
2. 2^nd^ digit is the longest digit (2>1>3>4>5)
3. 1^st^ and 2^nd^ digits are equal length, and longer than the remaining (1=2>3>4>5)


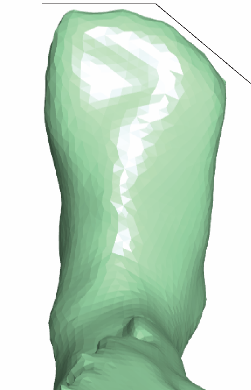

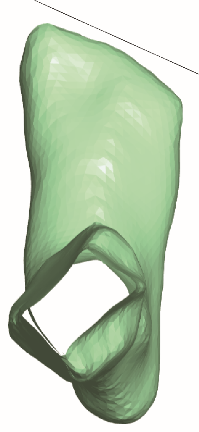

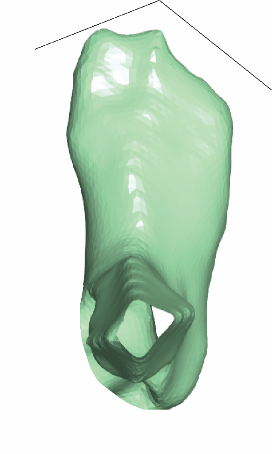


1. (2) (3)
